# Supplementary material for: Polyketide synthase gene clusters in the ericoid mycorrhizal fungus Oidiodendron maius: genome-wide prediction, expression profiling, and functional characterization of knockout mutants
Source: Front Plant Sci. 2026 Sep 16;17:1924430. doi: 10.3389/fpls.2026.1924430 (PMC13623903; doi:10.3389/fpls.2026.1924430)
Supplement: Supplementary file 1 [file SupplementaryFile1.zip › Supplementary Materials/Supplementary Folder 1.zip!/Oidma1_AssemblyScaffolds_Repeatmasked/knownclusterblast/cluster2/jgi.p_Oidma1_111044_mibig_hits.html]

| MIBiG Protein | Description | MIBiG Cluster | MiBiG Product | % ID | % Coverage | BLAST Score | E-value |
| --- | --- | --- | --- | --- | --- | --- | --- |
| AFT91368.1 | hypothetical\_protein | BGC0000338 | Echinocandin\_B\_biosynthetic\_gene\_cluster | 28 | 96.8692449355 | 171 | 2e-42 |
| AAS90013.1 | OrdA | BGC0000007 | Aflatoxin\_biosynthetic\_gene\_cluster | 28 | 98.7108655617 | 171 | 3.4e-42 |
| AAS90105.1 | OrdA | BGC0000006 | Aflatoxin\_biosynthetic\_gene\_cluster | 27 | 97.2375690608 | 164 | 4.2e-40 |
| AAS90081.1 | OrdA | BGC0000010 | Aflatoxin\_biosynthetic\_gene\_cluster | 27 | 97.2375690608 | 164 | 4.2e-40 |
| BAE71330.1 | oxidoreductase\_A;oxidoreductase/cytochrome\_P450\_monooxygenase | BGC0000004 | Aflatoxin\_biosynthetic\_gene\_cluster | 27 | 97.7900552486 | 161 | 2.1e-39 |
| AAS90035.1 | OrdA | BGC0000008 | Aflatoxin\_biosynthetic\_gene\_cluster | 27 | 97.2375690608 | 161 | 2.1e-39 |
| AAS90061.1 | OrdA | BGC0000009 | Aflatoxin\_biosynthetic\_gene\_cluster | 27 | 93.1860036832 | 158 | 2.3e-38 |
| BAF09098.1 |  | BGC0000672 | Phytocassane\_/\_oryzalides\_biosynthetic\_gene\_cluster | 26 | 82.5046040516 | 104 | 3.9e-22 |
| Bcin12g06400.1 |  | BGC0000631 | Botrydial\_biosynthetic\_gene\_cluster | 35 | 30.7550644567 | 98 | 2.1e-20 |
| AAK53584.1 | trichodiene\_oxygenase | BGC0000931 | Trichothecene\_biosynthetic\_gene\_cluster | 34 | 34.6224677716 | 95 | 2.4e-19 |
| AAK33083.1 | putative\_cytochrome\_P450 | BGC0001277 | Nivalenol\_/\_deoxynivalenol\_/\_3-acetyldeoxynivalenol\_/ | 34 | 34.6224677716 | 95 | 2.4e-19 |
| BAF09099.1 |  | BGC0000672 | Phytocassane\_/\_oryzalides\_biosynthetic\_gene\_cluster | 33 | 32.044198895 | 94 | 3.1e-19 |
| EAL89314.2 | cytochrome\_P450\_monooxygenase,\_putative | BGC0000686 | Helvolic\_acid\_biosynthetic\_gene\_cluster | 26 | 34.8066298343 | 75 | 1.9e-13 |
| CAQ18836.1 | putative\_cytochrome\_P450 | BGC0000954 | Ajudazol\_biosynthetic\_gene\_cluster | 25 | 33.5174953959 | 59 | 1.4e-08 |
